# Thermal conductivity and thermal boundary resistance of nanostructures

**DOI:** 10.1186/1556-276X-6-288

**Published:** 2011-04-04

**Authors:** Konstantinos Termentzidis, Jayalakshmi Parasuraman, Carolina Abs Da Cruz, Samy Merabia, Dan Angelescu, Frédéric Marty, Tarik Bourouina, Xavier Kleber, Patrice Chantrenne, Philippe Basset

**Affiliations:** 1 INSA Lyon, CETHIL UMR5008, F-69621 Villeurbanne, France; 2 Université de Lyon, CNRS, F-69621 Villeurbanne, France; 3 Université Lyon 1, F-69621 Villeurbanne, France; 4 Université Paris-Est, ESYCOM, ESIEE Paris, BP 99, 2 bd Blaise Pascal, F-93162 Noisy Le Grand, France; 5 Université de Lyon 1 - LPMCN UMR5586, CNRS, F-69621 Villeurbanne, France; 6 Université de Lyon - MATEIS UMR5510, CNRS, INSA Lyon, Université Lyon 1, F-69621 Villeurbanne, France

## Abstract

We present a fabrication process of low-cost superlattices and simulations related with the heat dissipation on them. The influence of the interfacial roughness on the thermal conductivity of semiconductor/semiconductor superlattices was studied by equilibrium and non-equilibrium molecular dynamics and on the Kapitza resistance of superlattice's interfaces by equilibrium molecular dynamics. The non-equilibrium method was the tool used for the prediction of the Kapitza resistance for a binary semiconductor/metal system. Physical explanations are provided for rationalizing the simulation results.

**PACS:**

68.65.Cd, 66.70.Df, 81.16.-c, 65.80.-g, 31.12.xv

## Introduction

Understanding and controlling the thermal properties of nanostructures and nanostructured materials are of great interest in a broad scope of contexts and applications. Indeed, nanostructures and nanomaterials are getting more and more commonly used in various industrial sectors like cosmetics, aerospace, communication and computer electronics. In addition to the associated technological problems, there are plenty of unresolved scientific issues that need to be properly addressed. As a matter of fact, the behaviour and reliability of these devices strongly depend on the way the system evacuates heat, as excessive temperatures or temperature gradients result in the failure of the system. This issue is crucial for thermoelectric energy-harvesting devices. Energy transport in micro and nanostructures generally differs significantly from the one in macro-structures, because the energy carriers are subjected to ballistic heat transfer instead of the classical Fourier's law, and quantum effects have to be taken into account. In particular, the correlation between grain boundaries, interfaces and surfaces and the thermal transport properties is a key point to design materials with preferred thermal properties and systems with a controlled behaviour.

**Figure 1:**
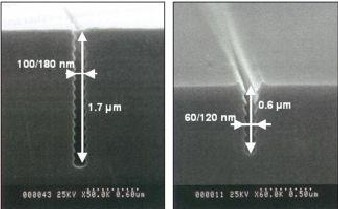
**SEM pictures obtained by the group ESYCOM and ESIEE at Marne-la-Vallee, France, showing two submicron trenches in a silicon wafer**.

In this article, the prediction tools used for studying heat transfer in low-cost superlattices for thermoelectric conversion are presented. The technology used in the fabrication of these superlattices is based on the method developed by Marty et al. [1,2] to manufacture deep silicon trenches with submicron feature sizes (Figure 1). The height and periodicity of the wavelike shape of the surfaces can be monitored. When the trenches are filled in with another material, they give rise to superlattices with rough interfaces. This was the motivation for studying both the thermal conductivity and the Kapitza resistance [3] of superlattices with rough interfaces. We focus mostly at the influence of interfacial width of the superlattices made of two semiconductor-like materials, with simple Lennard-Jones potential for the description of interatomic forces. Simulations of the Kapitza resistance for binary system of silicon with metal are also presented. These interfaces are difficult to be modelled, first of all because of the phonon-electron coupling that occurs at these interfaces and secondly because of the plethora of potentials which can be used. The choice of potential is based in a comparison of their performance to predict in a correct manner, the harmonic and anharmonic properties of the material. Results on the Kapitza resistance of a silver/silicon interfaces are also presented.

### Fabrication process of superlattices

**Figure 2:**
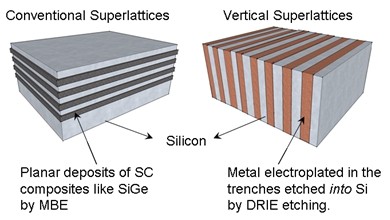
**Structural comparison between conventional superlattices and vertical superlattices**.

To reduce the processing time and the manufacturing costs, vertical build superlattices are proposed as opposed to conventional planar superlattices. In Figure 2, a schematic representation of the two types of superlattices is given comparing their geometries. With this process, silicon/metal superlattices can be fabricated. Although final device will have material layers in the tens of nanometre range, 5- and 15-μm width superlattices are fabricated using typical UV lithography. These thick layer superlattices are necessary to develop an accurate model of thermal resistance at the metal/semiconductor interfaces.

**Figure 3:**
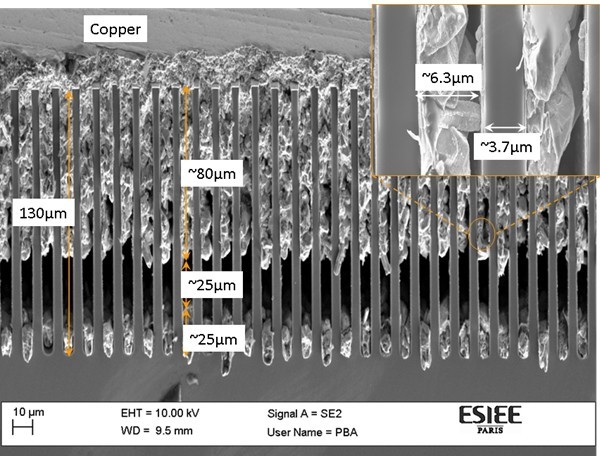
**SEM image of copper-filled 5-μm-wide trenches**.

Vertical superlattices were obtained by patterning and then etching the silicon by deep reactive ion etching (DRIE). The trenches were filled using electrodeposition on a thin metallic seed layer. In Figure 3, a scanning electron microscope (SEM) image of a processed silicon wafer with micro-superlattices is given. There are voids at the bottom of the trenches which are explained by the absence of the seed layer at the bottom, and the fact that they prevent any copper growth. These voids were successfully eliminated by increasing the amount of seed layer sputtered in subsequent trials. The excess copper on top, resulting from the trenches being shorted to facilitate electroplating, was polished away using chemical-mechanical polishing. This is done to electrically isolate the trenches from one another so as to allow thermo-electrical conversion.

We aim to fabricate optimized vertical nano-superlattices (with layers ranging <100 nm each) with high thermoelectric efficiency. High thermoelectric efficiency occurs for high electrical conductivity and low thermal conductivity. The electronic conductivity will be controlled though the Si doping and the use of metal to fill in the trenches. The film thickness needs to be decreased, to decrease the individual layer thermal conductivity and increase the influence of the interfacial thermal resistance.

To obtain such dimension on a large area at low cost, we are developing a process based on the transfer by DRIE of 30-nm line patterns made of di-block copolymers [4]. For this purpose, it is required to characterize them to the best possible degree of accuracy. Measurements at this scale will possibly be plagued by quantum effects [5,6]. That is the reason why we fabricated first micro-scale superlattices, to make thermoelectric measurements free from quantum effects and then applied the method to characterize the final nano-superlattice thermoelectric devices.

### Simulations: thermal conductivity of superlattices

When the layer thickness of the superlattices is comparable to the phonon mean free path (PMFP), the heat transport remains no longer diffusive, but ballistic within the layers. Furthermore, decreasing the dimensions of a structure increases the effects of strong inhomogeneity of the interfaces. Interfaces, atomically flat or rough, impact the selection rules, the phonon density of states and consequently the hierarchy or relative strengths of their interactions with phonons and electrons. Thus, it is important to study and predict the heat transfer and especially the influence of the height of superlattice's interfaces on the cross and in-plane thermal conductivities. This is a formidable task, from a theoretical point of view, as one needs to account for the ballistic motion of the phonons and their scattering at interfaces. Molecular dynamics is a relatively simple tool which accounts for these phenomena, and it has been applied successfully to predict heat-transfer properties of superlattices.

Two routes can be adopted to compute the thermal conductivity, namely, the non-equilibrium (NEMD) [7] and the equilibrium molecular dynamics (EMD) [8]. In this article, we have considered both methods to characterize the thermal anisotropy of the superlattices. In the widely used direct method (NEMD), the structure is coupled to a heat source and a heat sink, and the resulting heat flux is measured to obtain the thermal conductivity of the material [9,10]. Simulations are held for several systems of increasing size and finally thermal conductivity is extrapolated for a system of infinite size [11,12]. The NEMD method is often the method of choice for studies of nanomaterials, while for bulk thermal conductivity, particularly that of high conductivity materials, the equilibrium method is typically preferred because of less severe size effects. Comparisons between the two methods have been done previously, concluding that the two methods can give consistent results [13,14]. Green-Kubo method for nanostructures is proven to have greater uncertainties than those of NEMD, but a correct description of thermal conductivity with EMD is achieved by establishing statistics from several results, starting from different initial conditions.

The superlattice system under study is made of superposition of Lennard-Jones crystals and fcc structures, oriented along the [001] direction. The molecular dynamics code LAMMPS [15-17] is used in all the NEMD and EMD simulations. The mass ratio of the two materials of the superlattice is taken as equal to 2, and this ratio reproduces approximately the same acoustic impedance difference as that between Si and Ge. Periodic boundary conditions are used in all the three directions. Superlattices with period of 40*a*_0 _are discussed, where *a*_0 _is the lattice constant. The shape of the roughness is chosen as a right isosceles triangle. The roughness height was varied from one atomic layer (1 ML = 1/2*a*_0_) to 24*a*_0_. For each roughness, heat transfer simulations with NEMD were performed for several system sizes in the heat flux direction to extrapolate the thermal conductivity for a system of infinite size [11]. For EMD simulations, the size of the system is smaller than with NEMD simulations and only one size is considered 20*a*_0 _× 10*a*_0 _× 40*a*_0_, where the last dimension is perpendicular to interfaces.

**Figure 4:**
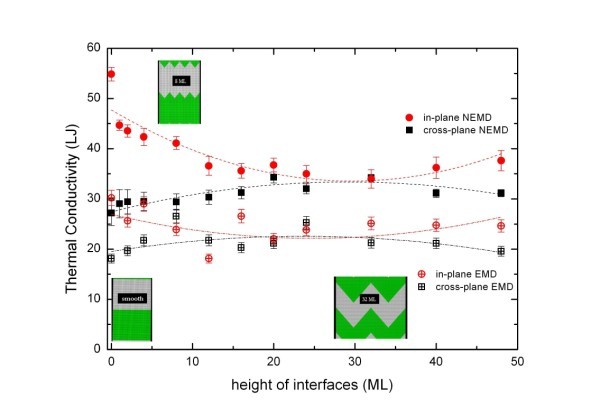
**Cross-plane and in-plane thermal conductivity functions of the height of interfaces calculated by EMD and NEMD methods**.

In Figure 4, we gathered the results for the in-plane and cross-plane thermal conductivities obtained by the two methods. The thermal conductivity is measured here in Lennard-Jones units (LJU), which correspond in real units typically to W/mK. At the low temperatures considered (*T *= 0.15 LJU), the period of the superlattice is comparable to that of the PMFP. The qualitative interpretation of the results shows that the thermal contact resistance of the interface has a strong influence on the superlattice thermal conductivity. The results previously obtained by NEMD method [12], and, in particular, the existence of a minimum for the in-plane thermal conductivity are now confirmed using the EMD method. The evolution of the TC as a function of the interfacial roughness is found to be non-monotonous. When the roughness of the interfaces is smaller than the superlattice's period, the in-plane thermal conductivity first decreases with increasing roughness. It reaches a minimum value which is lower by 35-40% compared to the thermal conductivity of the superlattice with smooth interfaces. For larger roughness, the thermal conductivity increases. The initial decrease of the in-plane thermal conductivity is quite intuitive if one considers the behaviour of phonons at the interfaces, which may be described by two different models. In the acoustic mismatch model [18,19], the energy carriers are modelled as waves propagating in continuous media, and phonons at the interfaces are either transmitted or specularly reflected. For atomically smooth interfaces, it is assumed that phonons experience mainly specular scattering. The roughness enhances diffuse scattering at the interface in all space direction.

In the diffuse-mismatch model, on the other hand, phonons are diffusively scattered at interfaces, and their energy is redistributed in all the directions [20]. In practice, the acoustic model describes the physics of interfacial heat transfer at low temperatures, for phonons having large wavelengths, while the diffuse model is relevant for small wavelengths phonons. At the considered temperature in the current study, we are most probably in an intermediate situation where the physics is not captured by one single model. Nevertheless, both models predict that a moderate amount of interfacial roughness will tend to decrease the in-plane TC, because rough interfaces will increase specular reflection and diffusive scattering of phonons travelling in the in plane direction. However, if the roughness is large enough, then locally, the phonons encounter smooth-like interfaces, and the partial group of phonons that are diffusely scattered in all space direction decreases. This might explain the further increase of the thermal conductivity when the roughness is large enough.

The behaviour of the cross-plane thermal conductivity is different: it increases monotonously with the interfacial roughness. For smooth interfaces, the cross-plane thermal conductivity is 50% lower than the in-plane thermal conductivity. This anisotropy has to be taken into account for thermal behaviour of systems made of sub-micronic solid layers. Invoking again the acoustic mismatch model, we conclude that the transmission coefficient of the solid/solid interface is smaller than the reflection coefficient, which is not surprising if we consider the acoustic impedance ratio of the two materials. Roughness increases the transmission coefficient as it increases the diffused scattering at the interface [12].

The same qualitative trend regarding the influence of the roughness on the thermal conductivity of superlattices has been reported previously for materials with diffusive behaviour, without thermal contact resistance [21]. In this case, the variation of the in-plane and cross-plane conductivities with the interfacial roughness is due to the heat flux line deviation that minimizes the heat flux path in the material that has the lower thermal conductivity. This tends to increase the cross-plane thermal conductivity. On the other hand, the increase of the roughness leads to the heat flux constrictions that decrease the in-plane thermal conductivity. The qualitative interpretation of the results shows that the thermal contact resistance of the interface has a strong influence on the superlattice thermal conductivity.

### Simulations: Kapitza resistance

#### Superlattices with rough interfaces

The discussion above shows that obviously the phononic nature of the energy carriers has to be taken into account to understand heat transfer in superlattices, and that the evolution of the superlattice TC may be qualitatively understood in terms of interfacial or Kapitza resistance. At a more quantitative level, the Kapitza resistance is defined by

and thus quantifies the temperature jump Δ*T *across an interface subject to a constant flowing heat flux *J*. In general, the Kapitza resistance may be computed using NEMD simulations by measuring the temperature jump across the considered interface. For superlattices, however, the direct method can be used only to measure easily the Kapitza resistance only for smooth surfaces, because of the difficulty involved in measuring locally the temperature jump for non-planar interfaces. To compute the Kapitza resistance for superlattices with rough interfaces, we have used EMD simulations, and the relation between *R*_K _and the auto-correlation of the total flux *q*(*t*) flowing across an interface:

where *S *is the interface area. The latter formula expresses the fact that the resistance is controlled by the transmission of *all *the phonons travelling across the interface.

In the situation of interest to us here, the transmission of phonons is expected to be strongly anisotropic, and thus the resistance developed by an interface should depend on the main direction of the heat flux. To measure this anisotropy, we have generalised the previous equation and introduced the concept of directional resistance, by considering the heat flux *q*_θ_(*t*) in the direction θ in (0,π/2) with the normal of the interface.

The resistance in the direction θ may be then quantified by the generalised Kapitza resistance:

This angular Kapitza resistance quantifies the transmission of the heat flux in the direction making an angle θ with the normal of the interface.

**Figure 5:**
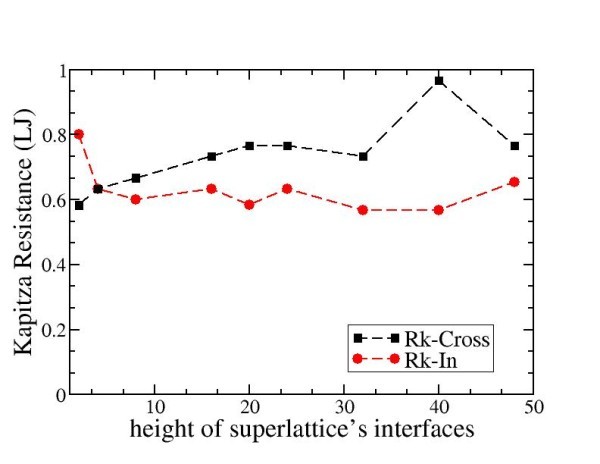
**Kapitza resistance function of the height of superlattice's interfaces**.

Figure 5 displays the generalised Kapitza resistances measured with MD for superlattices with rough interfaces having variable roughnesses. Again, the results are displayed in LJU, which correspond to a resistance of 10 × 10^-9 ^m^2^K/W in SI units. The period of the superlattice considered is larger than the PMFP, which here is estimated to be around 20*a*_0_. We have focused on two peculiar orientations θ = 0 and θ = π/2 which correspond, respectively, to the cross-plane and in-plane directions of the superlattices. It is striking that, for a given interfacial roughness, the computed resistance depends on the orientation θ. We have found that for almost all the systems analysed, the Kapitza resistance is larger in the cross-plane direction than in the direction parallel to the interfaces. This is consistent with the observation that the thermal conductivity is the largest in the in-plane direction (Figure 4). Again, this reinforces the message that the heat transfer properties of superlattices are explained by the phononic nature of the energy carriers, and that theses energy carriers feel less friction in the in-plane direction than that in the direction normal to the interfaces. Measuring the directional Kapitza resistance is a first step towards a quantitative measurement of the transmission factor of phonons depending on their direction of propagation across an interface.

#### Silver/silicon interfaces

The Cu and Ag films on Si-oriented substrates are the principal combinations in large-scale integrate circuits. Furthermore, with the fabrication process of vertical-built superlattices described in previous section, we are interested in the heat transfer phenomena related to the metal/semiconductor interfaces. The prediction of heat transfer in these systems becomes challenging when the thickness of the layers reaches the same order of magnitude as the PMFP. For heat transfer studies, MD is well suited for dielectrics since only phonons carry heat. For metals, coupling between phonons and electrons can be modelled with the two-temperature model [22]. For the above systems, it has been proven that the Kapitza resistance is mainly due to phonon energy transmission through the interfaces [23,24]. The interfacial thermal resistance, known as the Kapitza resistance [25,26] is important to be studied as it might become of the same order of magnitude than the film thermal resistance. In this section, interatomic potentials for Ag and Si are discussed. Using NEMD simulations, for an average temperature of 300 K, the Kapitza resistance of Si/Ag systems is determined.

Modified embedded-atom method (MEAM) is the only appropriate potential that can be used for metal/semiconductor systems. The first nearest-neighbour MEAM (1NN MEAM) potential by Baskes et al. [27] and the second nearest-neighbour MEAM (2NN MEAM) by Lee [28] are examined in the current study. The general MEAM potential is a good candidate for simulating the dynamics of a binary system with a single type of potential. For example, it can be applied for both fcc and bcc structures. Furthermore, this potential includes directional bonding, and thus can be applied for Si systems.

**Figure 6:**
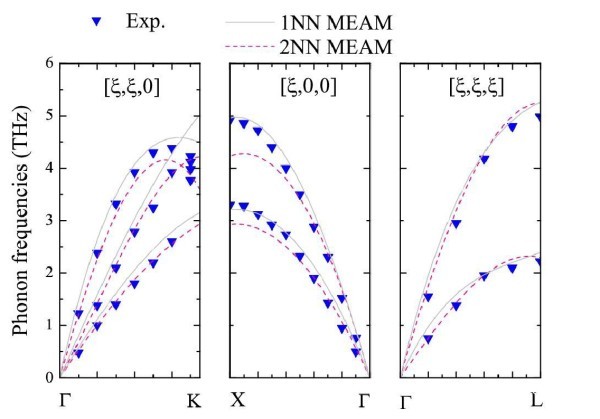
**Phonon dispersion curves using the potentials of 1NN MEAM, and 2NN MEAM for Ag**.

In dielectric materials heat transfer depends mainly on phonons' propagation and their interactions. To make the best choice among a great number of potentials for calculating thermal conductivity, the dispersion curves and the lattice expansion coefficient were studied. Electron transport predominates at the heat transfer in metals. MD cannot simulate electron movement, although some models are suggested in the literature to include the interactions between electron and phonons but without yet a satisfying results for investigating heat transfer. As it is not possible to test the quality of electronic interactions, only the lattice properties are commented to determine the correct potential for simulating Ag. The dispersion curves in the [ξ, 0, 0], [ξ, ξ, 0] and [ξ, ξ, ξ] directions are determined and compared with the experimental dispersion curves of Ag [29] for the 1NN MEAM and 2NN MEAM (Figure 6).

**Figure 7:**
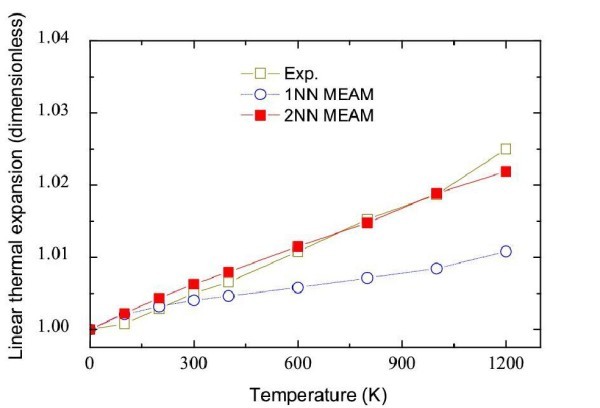
**Linear thermal expansion for Ag using 1NN MEAM and 2NN MEAM potentials**.

To compare the anharmonic properties of Ag, the equilibrium lattice parameter is simulated for different temperatures using the 1NN MEAM, and 2NN MEAM potentials. This is modelled with an fcc slab consisting of 108 atoms of silver with periodic boundary conditions in all the directions. Initially, the temperature of the crystal was 0 K. For each temperature the simulations are performed with a 20 ps constant-pressure simulation (NPT) during which the volume of the box occupied by the atoms for each temperature is stored. The mean value of the volumes of the equilibrated energy is used to calculate the linear expansion coefficient. For each constant temperature, the volume of the simulation box is divided by the volume at 0 K. This ratio is directly proportional to the expansion coefficient. The expansion coefficients of Ag, obtained for the two potentials are compared to the experimental values [30] in Figure 7. The uncertainties on the linear expansion coefficient variation are less than 5% compared with the experimental values.

The 2NN MEAM potential allows recovering the expansion coefficient for Ag quite accurately while the 1NN MEAM potential significantly underestimates it. For Ag, the two potentials provide a good description for the more basic properties, such as cohesive energy, lattice parameters and bulk modulus [31]. Even if the 1NN MEAM potential gives results closer to the experimental values for dispersion curves, the values obtained for the linear thermal expansion are not reasonable. Therefore, the 1NN MEAM potential cannot be considered appropriate for simulating heat transfer for silver. Regarding the investigation of heat-transfer temperature, the 2NN MEAM gives the best results for harmonic and anharmonic properties for silver and for silicon using the previous results of the literature [32]. Kapitza resistance is predicted for the 2NN MEAM Si/Ag potential. The interface thermal resistance, also known as Kapitza resistance, *R*_K_, creates a barrier to heat flux and leads to a discontinuous temperature, Δ*T*, drop across the interfaces.

**Figure 8:**
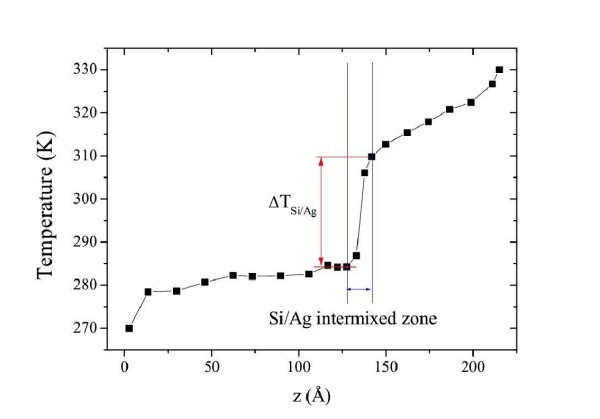
**Temperature profile for the Si/Ag system**.

The interactions between silicon and silver are described thanks to the 2NN MEAM potential in which the set of parameters has been determined to produce a realistic atomic configuration of interfaces. The model structure consists of two slabs in contact: one of Si with a diamond structure, and one of Ag. The periodic boundary conditions are used in all the directions and the Si crystal is composed of 7200 atoms, while the Ag crystal is composed of 2560 atoms. In the first stage of MD simulation, the system is equilibrated at a constant temperature of 300 K for 20 ps using an integration time step of 5 fs. The heat sources are placed in the extremes of the structure, and one layer of Si and Ag is frozen to block the movement of Si atoms in the *z*-direction. The temperature gradient is formed in the *z*-direction, imposing hot and cold temperatures above and below the fixed atoms in *z*-direction. Using an integration time step of 5 fs, the simulation is run for 5.0 ns, with an average system temperature of 300 K. In Figure 8, the temperature profile for the Si/Ag system is shown.

The Kapitza resistance obtained with NEMD is 4.9 × 10^-9 ^m^2^K/W. The temperature profile for Si is almost flat due its high thermal conductivity. With MD simulations, it is not possible to simulate heat transfer due to the electrons, and thus the steep slope of Ag is due to its low lattice thermal conductivity. The value *R*_KT _is in the range 1.4-125 × 10^-9 ^m^2^K/W which also includes the Kapitza conductance for dielectric/metal systems [33,34].

## Conclusions - Discussion

A new fabrication method for superlattices is used, reducing the time and fabrication costs. With the fabrication of vertical superlattices, several questions a rose for the influence of the roughness' height of the superlattices and the quality of interface on the thermal transport. When the length of the superlattice's period is comparable to the phonon-free mean path, the heat transfer becomes ballistic.

The cross-plane and in-plane thermal conductivities of a dielectric/dielectric (representing Si-Ge systems) superlattice are predicted using EMD and NEMD simulations. Both methods give the same tendencies for the anisotropic heat transfer at superlattices with rough interfaces. The in-plane thermal conductivity exhibits a minimum for a certain interfacial width, while the cross-plane thermal conductivity increases modestly in increasing the width of the interfaces. The Kapitza resistance of these interfaces is also studied, with a proposed methodology in this article, introducing the concept of directional thermal resistance. Values presented here are coherent with the difference between the in-plane and cross-plane thermal conductivities.

Molecular dynamics simulations are also used to study the metal/semiconductor interfaces. Among all the interatomic potentials that are available, the MEAM potential is a good alternative to work with since it can be used for different materials. At 300 K, the 2NN MEAM potential gives the best results for the fundamental properties associated with the heat transfer of silicon and silver. Previous results [23,24,32] suggest that interfacial thermal conductance depends predominantly on the phonon coupling between silicon and metal lattices so that Si/Ag can be simulated without considering the contribution of electron heat transfer. The value of magnitude of the Kapitza resistance for a Si/Ag system is within the range of Kapitza resistance proposed in the literature.

This study proves that making rough instead of smooth interfaces in superlattices is a useful way to decrease the thermal conductivity and finally to design materials with desired thermal properties. Furthermore, when more interfaces are added (rough or smooth), i.e. when the superlattice's period decreases, the interfacial thermal resistance becomes comparable to the superlattice's layers thermal conductivity. With these two parameters, namely, the introduction of rough interfaces and the decrease of the superlattice's period, we can create systems with controlled values of the thermal conductivity.

## Abbreviations

DRIE: deep reactive ion etching; SEM: scanning electron microscope; PMFP: phonon mean free path; NEMD: non-equilibrium molecular dynamics; EMD: equilibrium molecular dynamics; LJU: Lennard-Jones units;

## Competing interests

The authors declare that they have no competing interests.

## Authors' contributions

KT: Calculated the theoretical values for the thermal conductivity of super-lattices with NEMD and participated for the calculations of Kapitza resistance of the semiconductor superlattices with EMD method and drafted and revised the manuscript. JP: Participated in the design and fabrication (all steps) of the superlattices with micro- and nano-scale layers. CC: Calculated the Kapitza resistance of metal/semiconductor interfaces. SM: Calculated the Kapitza resistance of the semiconductor superlattices with EMD method and drafted the manuscript. DA: Participated in the development of the patterning of the "nano" superlattices using di-block copolymer. FM: Participated in the development of the high aspect ratio plasma etching of silicon for the "micro" and "nano" superlattices.TB: Participated in the development of the high aspect ratio plasma etching of silicon for the "micro" and "nano" superlattices. XK: Participated in the coordination. PC: Participated in the coordination and drafted and revised the manuscript. PB: Conceived and coordinated the COFISIS project, and also participated in the design of the superlattices and drafted and revised the manuscript.
